# Nonparametric tests for differential gene expression and interaction effects in multi-factorial microarray experiments

**DOI:** 10.1186/1471-2105-6-186

**Published:** 2005-07-21

**Authors:** Xin Gao, Peter XK Song

**Affiliations:** 1 Department of Mathematics and Statistics, York University, 4700 Keele Street, Toronto, ON M3J 1P3, Canada; 2 Department of Statistics and Actuarial Science, University of Waterloo, 200 University Ave. W., Waterloo, ON N2L 3G1, Canada

## Abstract

**Background:**

Numerous nonparametric approaches have been proposed in literature to detect differential gene expression in the setting of two user-defined groups. However, there is a lack of nonparametric procedures to analyze microarray data with multiple factors attributing to the gene expression. Furthermore, incorporating interaction effects in the analysis of microarray data has long been of great interest to biological scientists, little of which has been investigated in the nonparametric framework.

**Results:**

In this paper, we propose a set of nonparametric tests to detect treatment effects, clinical covariate effects, and interaction effects for multifactorial microarray data. When the distribution of expression data is skewed or heavy-tailed, the rank tests are substantially more powerful than the competing parametric F tests. On the other hand, in the case of light or medium-tailed distributions, the rank tests appear to be marginally less powerful than the parametric competitors.

**Conclusion:**

The proposed rank tests enable us to detect differential gene expression and establish interaction effects for microarray data with various non-normally distributed expression measurements across genome. In the presence of outliers, they are advantageous alternative approaches to the existing parametric F tests due to the robustness feature.

## Background

High density oligonucleotide microarray, spotted cDNA array, or other array technologies have presented not only daunting amount of expression data for biologists to explore the inherent biological mechanisms, but also challenging statistical analysis problems. A replicated microarray experiment involves multiple arrays to compare gene expression profile under different conditions. However, normality assumption justifying parametric testing is often untenable in microarray studies [1,2]. For instance, a set of 540 genes from a leukemia data set [3] were analyzed and various distributions for the different genes were found, in which only 13.3% genes have error distributions satisfying the normality assumption [4]. If the underlying distributions of expression measurements can be validated properly, model-based approaches such as likelihood or Bayesian inference can validly accept non-normally distributed data and gain satisfactory power to detect differentially expressed genes (e.g. [5-10]). For example, a hierarchical mixture-model has been proposed with parameterizations for Gamma or log-normally distributed measurements [10]. However when the distribution of the data is difficult to characterize, nonparametric inference makes less stringent distributional assumptions and thereby provide appropriate analysis.

Furthermore data contamination can arise in microarray setting due to different reasons. For instance, an image contamination can occur if a long scratch is present on the array image or a corner of the array is misaligned in the image processing stage. Sample contamination can occur if the mRNA sample is contaminated with other sources of RNA present in the laboratory environment. Such outliers are dramatically different from the majority of the observations and can greatly undermine the sensitivity of parametric approach. A method of assessing goodness of fit to a linear model has been used to automatically detect outliers that possess too large deviation from the overall pattern [11]. Alternatively, a quality index based on coefficient variation was adopted to filter out outlying values with poor quality [9]. Nevertheless the inspection process is time consuming for such large-scale expression data analysis [11]. In this context, nonparametric inference is advantageous as it is insensitive to the presence of outliers. Even without the step of outlier filtering, the validity and power of the nonparametric procedures would be minimally affected.

The development of both parametric and nonparametric methods to address the two condition problem in microarray setting has recently received much attention. Most of the parametric tests employed *t *or *t*-like statistics and differ primarily in the estimation of variance [12]. In contrast to these methods which treat the genes as separate fixed effects, the two-group Bayes method was proposed to treat the genes as arising from a certain population. Thus the dimensionality of the inference problem was reduced by sharing information across the array [5,9]. Nonparametric approaches have also been proposed for two-user defined groups [12,13]. The Wilcoxon rank sum test was considered in [14,15] to identify differentially expressed genes in comparison with the Fisher-Pitman permutation test, which is also referred as the nonparametric *t *test [15]. Recently, the Baumgartner-Weiß-Schindler test has been recommended to detect differentially expressed genes in two groups, which was shown to be less conservative and more powerful than the Wilcoxon rank sum test [16].

However, a microarray experiment often has more complicated design than that of two user-defined groups. Besides the treatment effects of interest, there may exist some clinical covariates such as age, gender and certain clinical symptoms, which also influence the gene expression level. For such experiments, a factorial design model is useful to account for the multiple sources of variation. Townsend and Hartl [6] derived a Bayesian model that has been widely used for the estimation of gene expression levels in multifactorial experiments [7,17]. This model has been extended [8] to accommodate not only additive error terms but also multiplicative error terms to resolve small yet statistically significant differences in gene expression. Alternatively, an overall ANOVA model has also been widely used that simultaneously considers all the genes on the arrays and incorporates array effect and dye effect [18]. A gene specific ANOVA model under the normality assumption was considered in [19]. A mixed linear model was proposed to assess gene significance in which both fixed treatment effects and random array effects were assumed [20]. Unfortunately, there has been no nonparametric procedure proposed up to date to analyze multifactorial microarray data. In addition, the establishment of interaction effect between the multiple attributing factors can help elucidate certain biological mechanisms related to the regulation of gene expression. Thus it is desirable to develop a set of nonparametric procedures to detect differential gene expression and establish interaction effects for multifactorial microarray data.

## Results

### Principle of the method

To account for the multiple sources of variation attributing to the gene expression, we consider the following model for each specific gene:

*X*_*kijn *_= *θ*_*k *_+ *T*_*ij *_+ *C*_*j *_+ *ε*_*kijn*_, *k *= 1,..., *K*; *i *= 1,..., *I*; *j *= 1,..., *J*; *n *= 1,..., *N *    (1)

with ∑_*j *_*C*_*j *_= 0, and ∑_*i*, *j *_*T*_*ij *_= 0, where *k *indexes for the gene number, *i *indexes for the treatment group, *j *indexes for the covariate group, *n *indexes for the replicate number. In the equation, *X*_*kijn *_represents the expression measurement, *θ*_*k *_represents the *k*^*th *^gene specific mean, *T*_*ij *_represents the effect of the *i*^*th *^treatment group (for instance, drug treatments, tissue types, and strains of mice) through its main effect and interaction effect with the *j*^*th *^level of the clinical covariate, and *C*_*j *_represents the effect of the *j*^*th *^level of the clinical covariate. The error terms *ε*_*kijn *_are independently and identically distributed random noise from a continuous distribution function *F*_*k*_.

To further discern the interaction effect, the treatment effect *T*_*ij*_, can be decomposed into *T*_*ij *_= *M*_*i*_+ *γ*_*ij *_with ∑_*i *_*M*_*i *_= 0 and ∑_*i*, *j *_*γ*_*ij *_= 0, where *M*_*i *_denotes the main effect of the treatment group and *γ*_*ij *_denotes the interaction effect. Interaction effects are often of biological interest when the treatment effects are heterogeneous across the levels of the clinical covariate. For example, consider a data set with mouse strains as treatment groups and tissue types as covariate groups, the interaction effects arise when the effects of different mouse strains are disproportional over different tissue types. It is worth noting that model (1) is related to ANOVA models proposed by other researchers [18-20]. The difference between our factorial model (1) and the existing ANOVA models is two-fold: model (1) accommodates multifactor effects on each specific gene, and it does not make normality assumption on the error terms *ε*_*ijkn*_.

To develop nonparametric rank tests for multifactorial microarray experiments, it is natural to consider rank procedure which can be viewed as nonparametric analogue of the parametric analysis of variance approach. The popular rank transform (RT) method consists of replacing the observations by their ranks in the combined sample and performing one of the standard analysis of variance tests on these ranks [21]. However in the general multifactorial model, the RT method is not valid for most of the common hypotheses due to the nonlinear nature of the rank transformation. For example in the presence of interaction effect, the naive application of ranks into ANOVA formula cannot be used to detect for main effect nor for the interaction effect. Theoretical validations of these limitations of RT method have been thoroughly discussed by Brunner and Neumann, Akritas, and Wilcox, among many others [22-28]. Since the RT method can be easily accomplished by using standard computer packages, extra caution needs to be exerted to prevent the inappropriate extensions of RT method for microarray data analysis under the multifactorial model. In the following, we shall present rank procedures which are similar to RT methods in the sense that they also resemble the analysis of variance approach, however they incorporate more rigorous treatment on the data rather than just replacing the actual observations by the overall rankings.

Usually as the first step of the analysis, we wish to assess whether the genes are differentially expressed among the treatment groups. The testing of treatment effect under model (1) is equivalent to the testing of the hypotheses: *H*_10 _: *T*_*ij *_= 0, for all *i*, *j *versus *H*_1*a *_: *T*_*ij *_≠ 0 for some *i*, *j*. To address this testing problem, we proposed to use the modified rank transform method (MRT) which consists of first standardizing the rank scores and then plugging them into the analysis of variance formula [25]. The resulting MRT statistic has proven to asymptotically follow a *χ*^2 ^distribution with (*I *– 1)*J *degrees of freedom. In a replicated microarray analysis, the sample size *N *is often so small that the large-sample asymptotic chi-squared distribution is not accurate enough to obtain valid *p*-values. To assess the significance of the rank statistic, the permutation method will be invoked to provide *p*-values of the observed statistic. An alternative way to reduce the computational burden encountered by the permutation procedure is to assess the significance of the proposed rank tests by the limiting chi-squared distribution. Table 1 provides the type I error rates of MRT based on the chi-squared approximation as the sample size increases from 5 to 15 and 20. It is demonstrated that a cell sample size of 20 or more are required for the chi-squared approximation to maintain type I error rate close to the correct nominal level.

**Table 1:** Convergence of type I error rates of the MRT test based on chi-squared approximation. The type I error rates of the MRT test based on chi-squared approximation were evaluated under varying sample sizes. A 2 × 2 design and a 3 × 4 design were considered.

Dist	Design	*N *= 5	*N *= 15	*N *= 20
*N*	2 × 2	0.077	0.047	0.052
	3 × 4	0.075	0.055	0.053

*U*	2 × 2	0.075	0.046	0.051
	3 × 4	0.083	0.055	0.053

*LN*	2 × 2	0.074	0.060	0.055
	3 × 4	0.078	0.061	0.056

*CN*	2 × 2	0.081	0.053	0.051
	3 × 4	0.072	0.054	0.053

*C*	2 × 2	0.072	0.046	0.052
	3 × 4	0.068	0.061	0.055

In practice, ties are commonly encountered in microarray data due to rounding and data modification [16]. In the presence of ties, we adopted the method of mid-ranks which assigns each tied individual the average of the tied ranks. There are other methods of dealing with ties such as the methods of randomization and the average statistics. However it has been shown that the randomization method is less powerful under the alternatives due to the supplementary random effects introduced by the randomization. In addition, the method of average statistics typically leads to a conservative test that has a lower significance level than the nominal one [29]. Thus the method of mid-ranks is most frequently used compared to other method to handle ties. As little is known about the small-sample performance of MRT using mid-ranks, it is of interest to conduct simulation studies to investigate this aspect. The related result is provided in the subsequent section.

An important aspect related to multifactorial design is to address treatment-covariate interaction effects. When the interaction is present, the gene expression level will be affected by the treatment disproportionally over different covariate levels. Based on the additive decomposition model for the treatment effects *T*_*ij *_= *M*_*i *_+ *γ*_*ij*_, the testing of interaction effect is equivalent to the testing of the hypotheses *H*_20 _: *γ*_*ij *_= 0, for all *i*, *j *versus *H*_2*a *_: *γ*_*ij*_≠ 0 for some *i*, *j*. As we have emphasized above, the RT method does not yield valid statistics for interaction effects (see [22-25]). Instead we employed the aligned rank transform test (ART) to test for the above hypotheses [30]. ART test consists of performing the analysis of variance test on the ranked residuals of the aligned observations. Although both utilize the ANOVA formula, the ART method differs from the RT method as it is based on residuals after the alignment. In contrast to RT, ART is a valid test for interaction regardless of the presence of main effects [31].

If there are no interaction effects, we can consider a simpler model: *X*_*kijn *_= *θ*_*k *_+ *M*_*i *_+ *C*_*j *_+ *ε*_*kijn*_, with *M*_*i *_denoting the treatment main effect and *C*_*j *_denoting the covariate effect. Testing for the treatment main effect corresponds to the hypotheses: *H*_30 _: *M*_*i *_= 0 for all *i *against *H*_3*a *_: *M*_*i*_≠ 0 for some *i*. We propose to employ the rank transform statistic suggested in [21]. It is worthy to point out that the testing of main effects in the absence of interaction is one of very few situations that naive application of the ANOVA formula on rank scores can yield valid statistic with satisfactory power properties.

In data analysis, the three testing procedures discussed above are connected. The following empirical rule regards how to proceed to choose the tests in a real data analysis. As the first step of analysis, the hypothesis of treatment effects (*H*_10_) is usually tested to see if the gene is differentially expressed across treatment groups. If *H*_10 _is accepted, no more actions will be taken as no differential expression is detected. If *H*_10 _is rejected, we may further perform the test for interaction effects (*H*_20_) to see if the differential expression is partly due to the interactions between treatment groups and covariate groups. The acceptance of *H*_20 _implies there exist no interaction effects. Then the testing for main effects (*H*_30_) can be pursued on the basis that the interaction effects are found insignificant.

### Single gene analysis

Simulation studies were conducted to evaluate the performances of the proposed rank methods in comparison with the other two competing methods, the parametric *F *test (FT) and the permutation *F *test (PFT) that uses the *F *statistic but computes *p*-values through permutations. The criterion used in the comparison is the efficiency gain relative to the FT method, defined as



where T can be either the MRT, ART, RT, or PFT. Obviously, when the test T outperforms the FT, the *EG *will be positive; otherwise, the *EG *is negative.

The performances of these methods were evaluated under different noise distributions and different numbers of replications. We considered a replicated factorial array experiment involving two treatment groups, two levels of a clinical covariate and varying cell sample sizes. Average type I error rates and power were calculated from 1,000 simulation runs. From the literature it has been shown that normal, uniform, log-normal, Cauchy and normal mixture distributions, among others, are commonly seen for microarray expression data [4]. In our simulation, we considered normal *N*(0, 1), uniform *U*(-2, 2), log-normal *LN*(0,1), Cauchy *C*(0.5) and contaminated normal *CN*(0.75, 0.5, 2) = 0.75*N*(0, 0.5) + 0.25*N*(0, 2). To some extent, contaminated normal can be used to model data with sample contamination, with one normal component representing the true underlying mRNA population of interest and the other normal behaving as the mRNA population from the contamination source. It is recognized that this normal mixture model may not be able to describe more irregular and dramatic data contamination such as distorted array image or scratched array regions. Fortunately the proposed nonparametric method does not rely on the correct characterization of the underlying distribution. This set of distributions were selected mainly for comparison purpose and they represent a broad range of characteristics from light-tailed to heavy-tailed, and from symmetric to asymmetric distributions.

We first evaluated the performance of the proposed MRT statistic for the testing of the treatment effects. We set the clinical effects as *C*_1 _= -0.5, and *C*_2 _= 0.5. Under the alternative situation, we set the treatment effects as *T*_11 _= 0.7, and *T*_12 _= 0.7, *T*_21 _= -0.9, *T*_22 _= -0.5, which were induced by the main effects *M*_1 _= 0.6, *M*_2 _= -0.6, and the interaction effects *γ*_11 _= 0.1, *γ*_12 _= 0.1, *γ*_21 _= -0.3, *γ*_22 _= 0.1.

Table 2 provides the results of the type I error and power of the MRT as well as its two competitors FT and PFT. The type I error rates of the FT appear around 0.05 in the case of light or medium-tailed distribution (normal and uniform). However for heavy-tailed distributions, especially in the case of Cauchy distribution, even with the sample size *N *= 10, the FT seems to be very conservative. Thus, the performance of the FT under the null can become rather poor if the error distribution is very different from the normal. In contrast, the type I error rates of MRT and PFT are advantageous as they are close to the correct nominal levels regardless of the underlying distribution.

**Table 2:** Type I error rates and power for treatments effects. The type I error rates and power were evaluated under five different error distributions – normal, uniform, lognormal, contaminated normal and Cauchy. The values inside and outside parenthesis are type I error rates and power, respectively. The *EG*(PFT) and *EG*(MRT) denote the efficiency gain of PFT and MRT versus FT.

Dist	N	FT	PFT	MRT	*EG *(PFT)	*EG *(MRT)
*N*	5	0.732 (0.050)	0.732 (0.050)	0.691 (0.054)	0.000	-0.056
	10	0.976 (0.050)	0.976 (0.050)	0.966 (0.050)	0.000	-0.010

*U*	5	0.575 (0.051)	0.565 (0.047)	0.486 (0.048)	-0.017	-0.155
	10	0.930 (0.054)	0.930 (0.054)	0.876 (0.050)	0.000	-0.058

*LN*	5	0.386 (0.029)	0.461 (0.047)	0.587 (0.048)	0.194	0.521
	10	0.576 (0.036)	0.620 (0.052)	0.889 (0.052)	0.076	0.543

*CN*	5	0.565 (0.039)	0.598 (0.058)	0.736 (0.055)	0.058	0.303
	10	0.829 (0.047)	0.838 (0.056)	0.958 (0.052)	0.010	0.156

*C*	5	0.230 (0.021)	0.366 (0.054)	0.564 (0.049)	0.591	1.452
	10	0.259 (0.016)	0.402 (0.052)	0.848 (0.055)	0.552	2.274

With regard to the power, the results are distribution dependent. For the medium or light-tailed distributions (normal and uniform), FT and PFT have similar performances and both of them achieve higher power than MRT test. In contrast, for the other distributions with heavy-tails, skewedness and contamination, MRT appears superior to the two competing methods. When the sample size *N *is 10, the MRT's efficiency gain, *EG*(*MRT*), is 54.3%, 15.6%, and 227.4% under log-normal, contaminated normal and Cauchy respectively. On the other hand, with the same sample size, the efficiency loss of the MRT is approximately 1.0% and 5.8% under normal and uniform. Compared to the amount of efficiency gain for the MRT versus the FT, the amount of efficiency loss seems to be marginal. Similar conclusions can be drawn when the sample size is 5. That is, the MRT's efficiency loss is approximately 5.6% for normal and 15.5% for uniform; the MRT's efficiency gain is 52.1%, 30.3%, and 145.2% for log-normal, contaminated normal and Cauchy respectively.

One interesting variant of the MRT method is the involvement of mid-ranks to handle ties. We randomly introduced *m *ties in the simulated data set. Table 3 lists the results of type I error and power of the three tests in the presence of *m *= 2, 5 or 10 pairs of ties. Comparing to Table 2, it is clear that these ties incurred only marginal differences in both type I error and power, even for the extreme scenario of *m *= 10. The slight increase of power in the presence of ties could be due to the decrease of within-group variation caused by averaging the ranks for tied observations.

**Table 3:** Type I error rates and power in the presence of ties. The type I error rates and power were evaluated under different error distributions and varying number of ties. The values inside and outside parenthesis are type I error rates and power, respectively. The cell sample size *N *= 5.

Dist	*# *of ties	FT	PFT	MRT
*N*	2	0.729 (0.051)	0.726 (0.051)	0.684 (0.052)
	5	0.730 (0.056)	0.730 (0.054)	0.682 (0.052)
	10	0.724 (0.053)	0.715 (0.051)	0.687 (0.051)

*U*	2	0.589 (0.054)	0.577 (0.052)	0.495 (0.052)
	5	0.580 (0.050)	0.568 (0.046)	0.493 (0.051)
	10	0.554 (0.048)	0.539 (0.046)	0.482 (0.048)

*LN*	2	0.391 (0.032)	0.455 (0.041)	0.581 (0.048)
	5	0.411 (0.039)	0.465 (0.054)	0.585 (0.056)
	10	0.412 (0.031)	0.448 (0.043)	0.589 (0.045)

*CN*	2	0.563 (0.038)	0.594 (0.051)	0.730 (0.053)
	5	0.575 (0.040)	0.599 (0.048)	0.735 (0.056)
	10	0.597 (0.044)	0.613 (0.054)	0.741 (0.057)

*C*	2	0.244 (0.023)	0.397 (0.057)	0.533 (0.052)
	5	0.262 (0.022)	0.370 (0.050)	0.562 (0.049)
	10	0.292 (0.030)	0.368 (0.048)	0.558 (0.055)

Next we examined the performance of the ART in testing for interaction effects, as well as the comparison to the FT and PFT. The simulation was set up as follows: under the null situation, the main effects were assigned, respectively, as *R*_1 _= -0.8 and *R*_2 _= 0.8, and *C*_1 _= -0.5 and *C*_2 _= 0.5; under the alternative situation, the main effects remained the same and additionally the interaction effects were given by *γ*_11 _= 0.6, *γ*_12 _= -0.6, *γ*_21 _= -0.6, and *γ*_22 _= 0.6. Table 4 provides the results of type I error and power of the three testing methods for the significance of the interaction effects. All these tests echo similar performances as presented in the above simulation study for the treatment effects. Notably, when the distribution of noise is heavy-tailed, skewed or contaminated, the ART appears considerably more powerful than the FT and the PFT. Regarding the testing of main effects, the RT statistic exhibits rather similar performance as the above two rank tests. The details of the RT are provided in the authors's website [32].

**Table 4:** Type I error rates and power of different tests for interaction effects. The type I error rates and power of the three different tests for interaction effects were evaluated under different error distributions. The values inside and outside parenthesis are type I error rates and power, respectively.

Dist	N	FT	PFT	ART	*EG *(PFT)	*EG *(ART)
*N*	5	0.711 (0.055)	0.697 (0.053)	0.698 (0.048)	-0.020	-0.018
	10	0.952 (0.051)	0.951 (0.051)	0.947 (0.051)	-0.001	-0.005

*U*	5	0.565 (0.049)	0.548 (0.045)	0.520 (0.050)	-0.030	-0.080
	10	0.891 (0.047)	0.891 (0.048)	0.833 (0.047)	0.000	-0.065

*LN*	5	0.405 (0.031)	0.430 (0.040)	0.579 (0.048)	0.062	0.430
	10	0.576 (0.038)	0.593 (0.045)	0.893 (0.050)	0.030	0.550

*CN*	5	0.525 (0.035)	0.540 (0.044)	0.718 (0.045)	0.029	0.368
	10	0.812 (0.043)	0.817 (0.050)	0.969 (0.051)	0.006	0.193

*C*	5	0.239 (0.014)	0.336 (0.029)	0.523 (0.044)	0.406	1.188
	10	0.274 (0.022)	0.364 (0.056)	0.859 (0.052)	0.328	2.135

### Global array analysis

The above discussion focuses on the single gene analysis. However, in microarray analysis the subsequent analysis step typically involves either adjusting the significance for multiple testing [33,34], or ranking genes according to the significance level such that the most relevant top *k *genes could be selected. Although discussing these global analysis approaches is beyond the scope of this paper, we are fully aware that the capability of a testing procedure to generate extreme *p*-values has a direct influence on the selection of the most relevant genes. When the Bonferroni procedure is employed to deal with the multiplicity, the Wilcoxon rank sum test is more conservative and less powerful than the Fisher-Pitman test or the parametric *t*-test [15]. It was further demonstrated that the discreteness of the exact permutation distribution of the Wilcoxon test is responsible for the conservatism [16]. Because of this, the Baumgartner-Weiß-Schindler test is recommended, as its exact permutation distribution has more non-zero mass probabilities and capable of generating richer small *p*-values than the Wilcoxon test. It is worthy pointing out that as the Bonferroni procedure is almost always more conservative than other multiple testing procedures, it will suffer most from the discreteness problem of the permutation distribution. Other multiple testing procedures impose less stringent *p*-value thresholds, therefore they are affected by the discreteness problem to a lesser extent.

**Figure 1:**
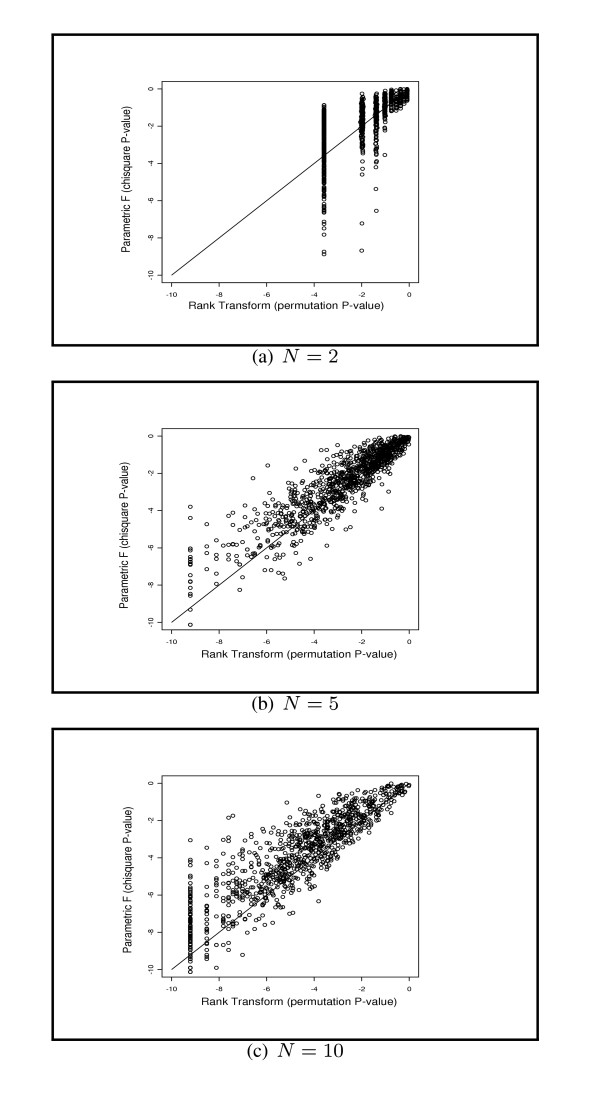
**Comparison of the MRT test vs the parametric FT test. **The *P*-values of the MRT test (X-axis) vs the FT test (Y-axis) under lognormal distribution were plotted under the logarithm scale. The replicate number ranges from 2, 5 to 10.

Our rank methods face the same issue as they use the permutation distribution to obtain *p*-values. It is crucial to examine how the discreteness of permutation distribution affects the performance of the MRT. We plotted the *p*-values (in log scale) of the MRT versus those of the FT in the connection to the first simulation study of testing for the treatment effects with 1000 runs. Figures 1a – 1c, corresponding to *N *= 2, 5, 10, depict the agreement between the MRT and FT tests under the log-normal noise, in which the perfect agreement is indicated by the solid 45° division line. We comment that (i) the symmetry around the 45° division line decreases as the number of replicates *N *increases, this implies that the MRT becomes more capable of producing extreme *p*-values than the FT test. Thus when *N *is 5 or larger, the permutation approach works reasonably well for the MRT method; and (ii) when *N *is small, say 2, the *p*-value of the MRT is often bounded due to the limited number of distinct probability mass points. For the example of the 2 × 2 design, as the permutation is carried out within each covariate group, the number of different permutation configurations equals . With *N *= 2, the number of possible different permutations is limited to only 36, so there are at most 36 different probability mass points. When *N *increases to 5, the resulting number of permutations increases to 6.35 × 10^4^, which considerably alleviates the problem of discreteness and improve the performance of the MRT. When *N *increases to 10, the corresponding number of permutations increases to 3.41 × 10^10^, and consequently further lessens the discreteness problem.

**Figure 2:**
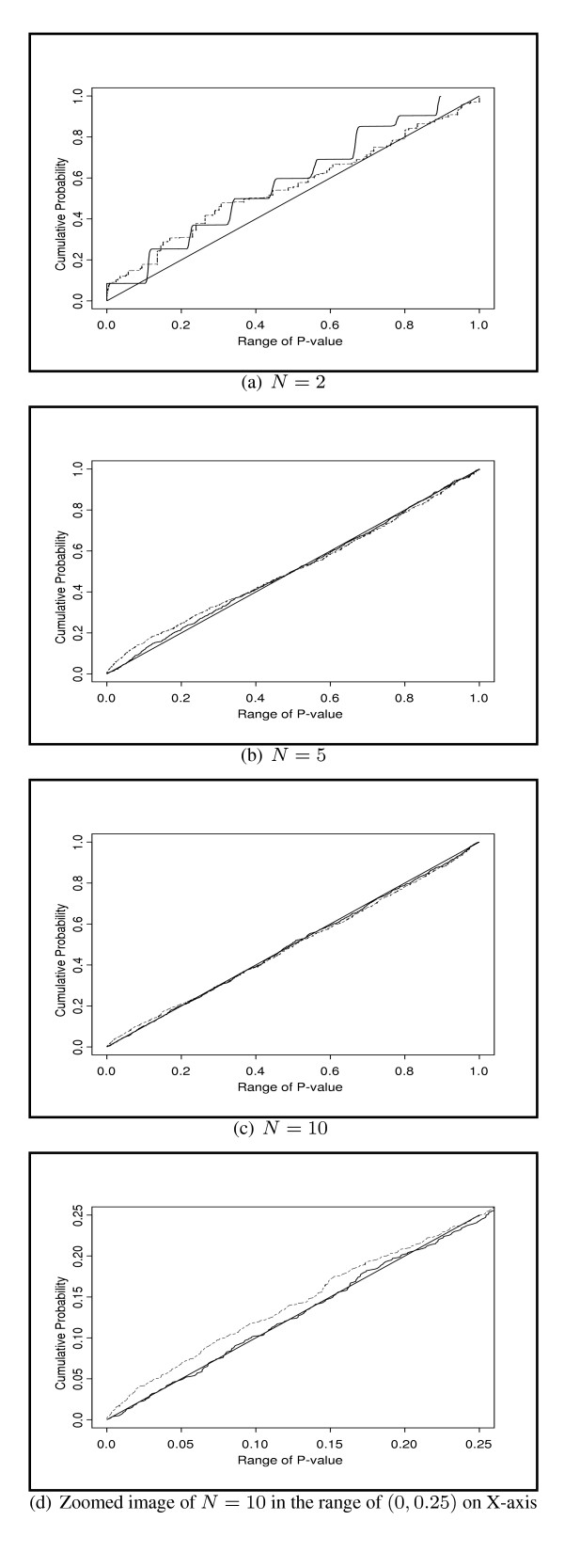
**The empirical CDFs of the *P*-values of the MRT test.**The empirical cumulative distributions of the *P*-values of the MRT test obtained from the permutation method or the chi-squared approximation are compared with that of the true *p*-value under the null situation. The *y *= *x *line denotes the CDF of true *p*-values; solid curve denotes the CDF of permutation *p*-values; dashed curve denotes the CDF of chi-squared *p*-values. Figure 2d is the zoomed image of the Figure 2c into the *p*-value range of (0, 0, 25).

In order to fully understand the effect of the discreteness of the permutation distribution, it is of interest to compare the *p*-values from the permutation method to the *p*-values obtained from the true distribution. Under the null situation, it is known that the *p*-values of MRT obtained from the true distribution should follow a uniform distribution on (0, 1), and the corresponding cumulative distribution function (CDF) should be the straight line *y *= *x*, *x *∈ (0, 1). Figure 2a to 2d provide the comparisons of the empirical CDFs of the permutation *p*-values versus the CDF of the true *p*-values for MRT test under the null situation when *N *= 2, *N *= 5, and *N *= 10. It is observed that with *N *= 2, the CDF of permutation p-values appears as a step function due to the discreteness at the limited number of probability mass points. The overall curve does not match very well with the CDF of the true *p*-values. When the sample size increases to 5 and 10, the agreement between the CDF of permutation *p*-values and the CDF of true *p*-values greatly improves and majority of the two curves overlap with each other. Therefore the plots suggest that the discreteness problem of permutation *p*-values is almost diminished with sample sizes greater than 5. In addition, the CDFs of the *p*-values from the chi-squared approximation are plotted in these figures. It is shown that the discrepancy between the CDFs of the chi-squared *p*-values and true *p*-values is generally larger than that between the permutation *p*-values and the true *p*-values. For instance, the Figure 2d is a zoomed image of the Figure 2c into the *p*-value range of (0, 0.25), it is shown that the CDF of the chi-squared *p*-values falls high above the *y *= *x *line indicating large inflation of type I error rate, while the CDF of the permutation *p*-values matches well with the *y *= *x *line. In conclusion, the permutation method provides a better control of type I error rate and therefore is more preferable compared to the chi-squared approximation in small sample size scenario.

### Biological data analysis

**Figure 3:**
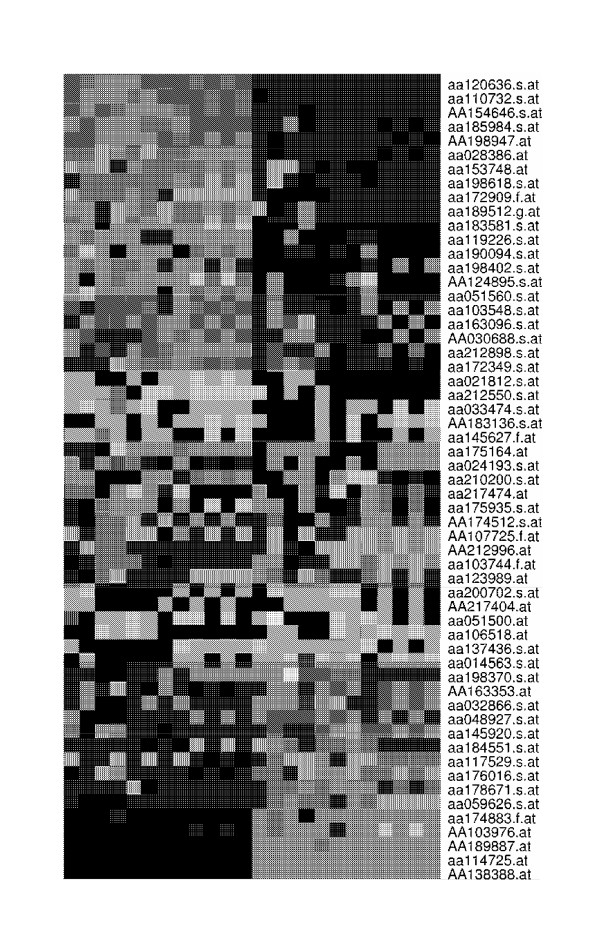
**The common list of genes identified by all the three methods.**The figure provides the common list of 57 genes identified by all the three methods in their top 100 rankings as differentially expressed in two mouse strains of the data of Sandberg et al. (2000).

We now illustrate the proposed MRT and ART methods as well as their competitors, the FT and the PFT methods, to analyze the gene expression data collected from six brain tissue regions in two mouse strains [35]. The data is obtained from [36], which contains a subset of 1000 genes. The purpose of the study was to investigate the genetic components contributing to the neurobehavioral differences between two mouse strains. For each mouse strain, the samples were obtained from 6 tissue regions, which can be viewed as a clinical covariate with 6 levels. For each mouse strain and a specific tissue, the expression profiles of two biological replicates were assessed. The *p*-values of the MRT versus respective *p*-values of the FT and the PFT were plotted and the MRT appears to be less capable of producing extreme *p*-values than the FT due to the low replicate numbers. In fact, this discreteness phenomenon has been unveiled in the simulation study through Figure 1.1. It was further shown that for the majority of genes the MRT and the PFT agree with each other. Among the top 100 genes selected by the MRT, 61 genes were selected by the FT and 77 genes were selected by the PFT. We then selected 57 genes that were identified as differentially expressed in two mouse strains by all the three methods in their top 100 rankings. To verify if these selected biomarkers really play any biological roles in the neurological phenotypic differences in mouse strains, we explored the gene functions by NetAffx Analysis Center in Affymetrix website [37]. The complete list of the functions of these 57 genes are available from the authors' website. Among these 57 genes, 24 genes share similar functions related to protein binding, transfer activity, signal pathway, receptor activities and mitochondrial electron transport chain, which are known to be essential to the function of nervous system. Another 14 genes share similar functions related to muscle movement, catalytic activity, kinase activity, hydrolase activity, and two other genes are related to hormone regulations, which are all related to the proper function and the regulation of nervous system. In total, 40 genes out of our list of 57 genes exhibit biological functions attributing to the phenotypic difference in the two mouse strains. Figure 3 displays that the selected 57 genes yield a clear separation of the samples from the two mouse strains. Therefore, the common list of genes identified by these three methods provides a reliable list of biomarkers.

If more exploratory research can be afforded to look for other genes, it is suggested to investigate the genes identified exclusively by the MRT (not by either the FT or the PFT). This extra list might provide a potential list of candidate genes that did not pass the two *F*-tests due to non-normal distributed noise in the data. To scrutinize this list, we also investigated the functions of 19 genes remaining in the list. The information regarding these 19 genes' functions is also available from the website as above. Among these 19 genes, 11 genes share similar functions as protein binding, transfer activity, signal pathway, receptor activities, mitochondrial electron transport chain, catalytic activities and kinase activities. It remains inconclusive if the other 8 genes can be supported as true positives due to the lack of known biological evidence.

As selecting the top listed genes only provides the set of most favorable candidates, no probabilistic statement can be attached to the findings. Alternatively, we can assess the significance of the findings under the multiple testing framework. Instead of using the stringent Bonferroni procedure, we applied the Benjamini and Hochberg's linear step-up procedure to control false discovery rate (FDR) [38]. As this procedure selected genes based on the ordered *p*-values, the significant genes were chosen consecutively down the top gene lists. By Controlling the FDR at level 0.05, the parametric F-test found 13 significant genes, 8 of which were found by either the permutation *F*-test or the nonparametric rank test. Given that there are only two replicates in the data set, it is not surprising that permutation-based methods identified a smaller number of significant genes, due to the discreteness of the permutation distribution discussed above.

**Figure 4:**
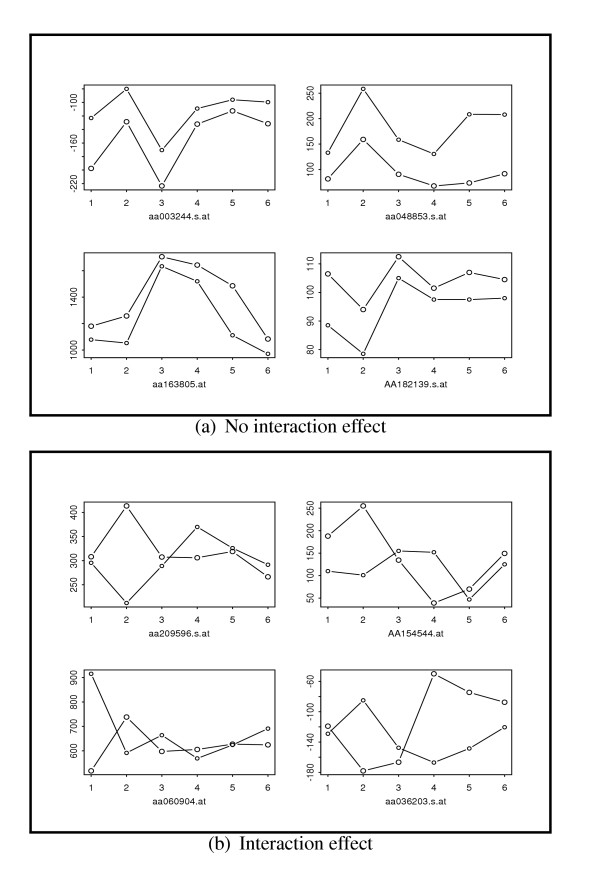
**Comparison of genes with and without interaction effects.**For each specific gene, two expression profiles are plotted for each of the two mouse strains across six brain regions-amygdala, cerebellum, cortex, entorhinalcortex, hippocampus and midbrain, which are denoted by 1 to 6 on x-axis. Figure 4a provides the expression profiles of four genes without interaction effects. Figure 4b provides the expression profiles of fours genes with interaction effects.

The interaction effect can arise when the effect of changing mouse strain is disproportional over different brain regions. The ART, FT and PFT were applied to test for the interaction effects between the mouse strains and the tissue regions. Comparison of the *p*-values from the ART versus the respective *p*-values from the FT and the PFT demonstrate a good deal of agreement among the three methods. Since the permutation was carried out on the basis of 24 aligned observations, the number of distinct permutations is so large that the discreteness problem is alleviated. Among the top 100 genes selected by the ART, 80 and 80 genes appeared in the top 100 rankings by the FT and the PFT, respectively. To visualize the interaction effects, for each gene the two profile curves for the two mouse strains were plotted representing the average expression levels over the six brain regions. Figure 4 provides examples of the profile curves of genes which are identified as having interaction effect by all the three methods versus genes which are found to have no interaction effects. For genes with no interaction effect, the two curves have parallel trends and differ by a vertical shift corresponding to the strain effect. In contrast, for genes with interaction effect, the two curves exhibit rather different patterns and even intersect with each other. For instance, the level of probe AA209596 was higher by two-fold in the cerebellum of strain 129SVEv compared with the C57BL/6 cerebellum. By contrast, in the entorhinalcortex region the level of probe AA209596 was lower by a factor of 1.2-fold in 129SVEv. Thus the differential expression between the two mouse strains reverses direction in two different brain regions. Probe AA209596 corresponds to gene TIMM13 which is translocase of inner mitochondrial membrane and has prominent expression in the large neurons in the brain. The TIM family plays important role in neurological behaviors as mutation of TIM gene is linked to neurobehavioral disorders such as deafness. Our finding suggests that the strain effects and brain region effects interact to regulate the expression of TIMM13. This analysis exemplifies how certain interacting mechanism behind gene expression can be unveiled via the interaction test on multifactorial microarray data.

## Discussion

Because there is a loss of information whenever the original data is collapsed to ranked data, the abandonment of parametric methods may not be cost-effective in all settings. In this article we have thoroughly investigated the positives and negatives of the proposed nonparametric rank tests versus the parametric ANOVA tests: (1) Due to the information loss, the rank tests are marginally less powerful than the ANOVA tests for normal, uniform or other light-tailed distributions. On the other hand, our simulation illustrated that the rank tests are substantially more powerful than the ANOVA tests if the data follow heavy-tailed, skewed or asymmetric distributions. (2) Our investigation also demonstrated that reasonable number of replicates (*N *≥ 5 for 2 × 2 design) are required to lessen the discreteness of permutation distribution encountered by the rank tests to evaluate *p*-values. In contrast, when the normality assumption is validated, the *p*-value of the parametric ANOVA statistic can be evaluated from the exact *F *distribution. (3) In the presence of severe outliers, the robust rank tests is more favorable than the parameter ANOVA tests. (4) When it is difficult to characterize the distribution of the data, the proposed distribution-free rank tests are useful to conduct an appropriate and powerful analysis.

**Figure 5:**
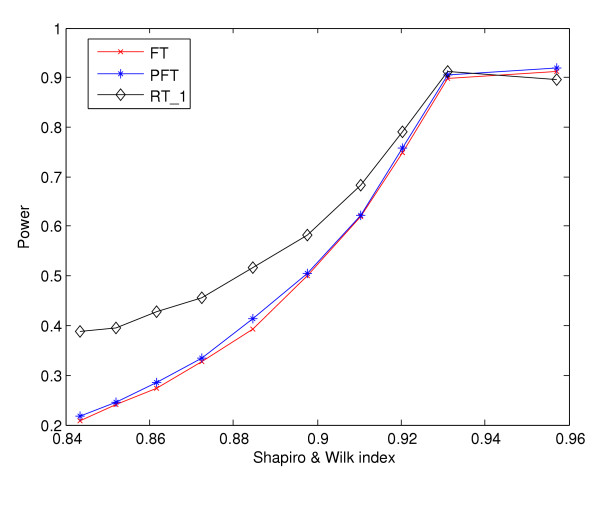
**Empirical power curves of three competing methods with respect to Shapiro-Wilk statistic. **The empirical power curves of the three competing methods – MRT, FT and PFT, are plotted against the varying level of Shapiro-Wilk statistic. The Shapiro-Wilk statistic is employed to assess the magnitude of the heavy-tailedness in the distribution.

As the comparative properties of rank tests relative to ANOVA tests are distribution dependent, distribution diagnostics can help the practitioners to determine which test will yield better power for a specific data set. Graphic inspections such as box-plot and normal probability plot offer a convenient way to visualize the shape of the underlying distribution. To quantify the magnitude of the deviation from normality, the Shapiro-Wilk test can be performed [39]. Let *x*_[1]_,...,*x*_[*N*] _be the ordered values of *N *independent and identically distributed observations. Let *z*_[1]_,...,*z*_[*N*] _denote the vector of the associated quantiles of the standard normal distribution. The Shapiro-Wilk statistic is defined as the squared correlation between the ordered data values (sample quantiles) and the normal quantiles:



For data that are really generated from normal distribution, the *W *statistic would be close to one. A smaller value of *W *indicates more deviation from normality. To further discern the deviation due to heavy-tail from the deviation due to light-tail, another statistic W* similar to the above Shapiro-Wilk statistic can be formed. The W* is defined as the correlation between the sample quantiles and the quantiles from a uniform distribution. As a result, the relative sizes of *W *and W* indicate the tail property for a given distribution. For instance, data generated from a heavy-tailed distribution would yield *W *> W*. This is because the correlation between a heavy-tailed distribution with the medium-tailed normal distribution should be stronger than that with the light-tailed uniform distribution. A reasonable threshold value *τ *for the statistic *W *will be determined by the comparative property of the nonparametric test relative to the two parametric F tests. A simulation-based approach can be invoked to numerically calculate this cutoff value. We illustrate such a procedure in a design model with *R *= 2, *C *= 2 and *N *= 5. The noise *ε*_*ijn *_were simulated from a normal distribution. Let *ε*_[1]_,..., *ε*_[20] _be the ordered noise. We gradually introduced heavy-tailedness into the data set by pulling the left and right end points of the ordered list of noise further away from the center. Each time a new data set was generated with  = *ε*_[*i*]_, for *i *= 4,..., 17, and  = *ε*_[*i*] _* *d*, for *i *= 1, 2, 3, 18,19, 20, and *d *was chosen from the varying range of 1.1, 1.3,..., 3. The corresponding *W *and the *p*-values of the FT, PFT and MRT tests were recorded for the data set. The result was summarized based on 1000 replications. In Figure 5, the empirical power curves of the three competing methods were plotted against the varying level of *W*. Our simulation demonstrates that as heavy-tailedness is introduced into the data set, *W *level decreases correspondingly. When *W *value is above 0.92, the two parametric methods outperform the nonparametric method. When *W *value is below 0.92, the nonparametric method is superior to the two parametric competitors. Thus for the specific design setting that we simulated, we choose a threshold value of *τ *= 0.92. If *W *<*τ *and *W *> W*, we would recommend the use of the nonparametric method. Among many sources of the normality violation discussed above, if *τ *= 0.92 was used as the cutoff, we found about 10% of genes in the data set of Sandberg et al. [35] whose expression measurements are from heavy-tailed distributions.

With regard to future extensions of the proposed methods, the tests discussed above can be applied to a high-way layout by collapsing these covariates into one. For example, a covariate with *J *levels and another covariate with *K *levels can be combined as a single factor of *JK *levels, so that the treatment effects can still be tested using the above two-way layout. When the data contains continuous covariates in certain applications, one can simply apply the proposed rank test method on the basis of residuals, the differences between the observations and the least squares fitted values calculated by using all the continuous covariates. Furthermore, it is possible to extend our methods to accommodate the dependence or heteroscedasticity which might occur in the microarray data sets. If the variances vary across different covariate groups, *j *= 1,..., *J*, the *MRT *statistic can still be employed to test for treatment effects using the standardized overall rank *Z*_*ijn *_[25]. To deal with two-way models with repeated measures on one factor or on both factors, the rank statistic can be extended to a quadratic form incorporating an estimated covariance matrix reflecting the dependence structure in the data [40].

In this article, we have focused on the interaction effects between multiple attributing factors to the gene expression. Currently there has been an increasing interest in studying interactions between genes as opposed to clinical factors. To address this problem, we could select a number of genes and treat their expressions measurements as explanatory variables. The biological phenotype of interest can be chosen as the response variable. Then a linear model can be fitted linking the gene expressions and biological phenotype. The aforementioned interaction tests can be applied to this setting to investigate the possible interactions among the genes.

## Conclusion

We have presented a set of nonparametric tests to detect treatment effects, clinical covariate effects, and interaction effects for multifactorial microarray data. These methods can be extended to accommodate high-way layouts, continuous covariates, dependent observations and heteroscedasticity which might occur in the microarray data sets. The proposed nonparametric procedures will prove to be of wide use in microarray data analysis as they can accommodate various noise distributions across genome.

## Methods

### Rank test for treatment effects

The first hypothesis *H*_01 _is formulated to test for treatment effects in two-way layout. Correspondingly, we have proposed a modified rank transform (MRT) test. This test standardizes the rank scores before plugging them into the analysis of variance formula. For simplicity in notation, we suppress the index *k*, as all the observations in the model are from a specific gene *k*. Let *R*_*ijn *_denote the rank of *X*_*ijn *_among all of the observations and define . Let  denote the sample variance of ranks within the j^*th *^column. Define the standardized rank score *Z*_*ijn *_= *R*_*ijn*_/*s*_*j*_. Denote the marginal and overall averages of the standardized rank scores by  and . The proposed modified rank transform statistic takes the following form:



It has been shown that the standardization procedure is essential for the validity of the MRT method as the nonlinear rank transformation introduces the heteroscedasticity into the ranked data [25]. To assess the significance of the rank statistic, the permutation method will be invoked to provide *p*-values of the observed statistic. In implementation, we randomly relabel *I *treatment groups within each of *J *covariate levels. Namely, the set of observations *X*_1*j*1_,..., *X*_1*jN*_,..., *X*_*Ij*1_,..., *X*_*IjN *_are shuffled within column *j *for 1 ≤ *j *≤ *J*. For illustration purpose, consider a microarray data set with the covariate consisting of six different tissue regions and the treatment consisting of two distinct mouse strains. The six covariate levels correspond to the six tissue regions. To generate a permuted data set, for the 2*N *measurements obtained from the same tissue region, we randomly assign *N *of them to the first mouse strain and assign the remaining observations to the second mouse strain. Repeat this procedure until we have permuted for all the tissue regions to generate a new permuted data set. Then we calculate the proportion of the resulting statistic (3) being equal to or larger than the observed statistic over 10,000 permutations to obtain the permutation *p*-value.

### Rank test for interaction effects

The second hypothesis *H*_02 _is formulated to test for interaction effects in two-way layout. To address this testing problem, the ART test is proposed to perform the analysis of variance test on the ranked residuals,  of the aligned observations .



Here  and  are the Hodges-Lehmann estimates of the two main effects given by



Again, with low replicates, we propose to use the permutation method to compute *p*-values under the null *H*_20_. In implementation, we randomly relabel both indices *i *and *j *within all the aligned observations and obtain the empirical *p*-value over 10,000 permutations.

### Rank test for main effects

The third hypothesis *H*_03 _is formulated to test for main effects in the absence of interaction effects in a two-way layout. We propose to employ the rank transform statistic suggested in [21] which is formulated as follows:



The resulting RT statistic asymptotically follows a *χ*^2 ^distribution with *I *– 1 degrees of freedom. Likewise, we can test if there is a difference of gene expression among the clinical covariate levels for gene *k*, using a test statistic similar to (4), with only indexes *I *and *J *being swapped.

## Authors' contributions

XG and PS developed the methods and wrote the manuscript.
